# Hyperpolarised ^13^﻿C MRI: a new horizon for non-invasive diagnosis of aggressive breast cancer

**DOI:** 10.1259/bjrcr.20190026

**Published:** 2019-05-15

**Authors:** Oshaani Abeyakoon, Arash Latifoltojar, Fiona Gong, Marianthi-Vasiliki Papoutsaki, Rafat Chowdhury, Matthias Glaser, Hassan Jeraj, Ramla Awais, Christopher Holt, Frazer Twyman, Erik Arstad, David G Gadian, David Atkinson, Arnaud Comment, James O'Callaghan, Lorna Smith, Teresita Beeston, Joey Clemente, Neill Patani, Rob Stein, Mariia Yuneva, Gyorgi Szabadkai, Steve Halligan, Shonit Punwani

**Affiliations:** 1 Department of Radiology, University College London Hospitals NHS Foundation Trust, London, UK; 2 Centre for Medical Imaging, Division of Medicine, University College London, London, UK; 3 Institute of Nuclear Medicine, University College London Hospitals NHS Foundation Trust, London, UK; 4 Department of Chemistry, University College London, 20 Gordon Street, London, UK; 5 Department of Oncology, University College London Hospitals NHS Foundation Trust, London, UK; 6 Pharmacy Department, University College London Hospitals NHS Foundation Trust, London, UK; 7 Institute of Child Health UCL Great Ormond Street, London, UK; 8 General Electric Healthcare, HP8 4SP, Chalfont St Giles, UK; 9 Department of Surgery, University College London Hospitals NHS Foundation Trust, London, UK; 10 The Francis Crick Institute, 1 Midland Road, London

## Abstract

Hyperpolarised ^13^C MRI (HP-MRI) is a novel imaging technique that allows real-time analysis of metabolic pathways *in vivo*.^1^ The technology to conduct HP-MRI in humans has recently become available and is starting to be clinically applied. As knowledge of molecular biology advances, it is increasingly apparent that cancer cell metabolism is related to disease outcomes, with lactate attracting specific attention. ^2^ Recent reviews of breast cancer screening programs have raised concerns and increased public awareness of over treatment. The scientific community needs to shift focus from improving cancer detection alone to pursuing novel methods of distinguishing aggressive breast cancers from those which will remain indolent. HP-MRI offers the opportunity to identify aggressive tumour phenotypes and help monitor/predict therapeutic response. Here we report one of the first cases of breast cancer imaged using HP-MRI alongside correlative conventional imaging, including breast MRI.

## Case presentation

A 40-year-old nulliparous female presented with a right breast lump. She had no known family history of breast or ovarian cancer. On examination there was a palpable area of indeterminate nodularity in the upper outer quadrant of the right breast. There was no palpable axillary lymphadenopathy.

## Investigations

Mammography, ultrasound, and dynamic contrast enhanced MRI were performed for diagnosis and staging. A HP-MRI study (as described below) was then performed prior to treatment. Ethical permission was granted (Research Ethics Committee (REC) reference number 17/LO/0431 ; ClinicalTrials.gov Identifier: NCT03687645 ) and written informed consent obtained.

A fluid-path was filled with [1-^13^C] pyruvate under aseptic conditions.^3^ The assembled path was loaded into a hyperpolariser unit (SPINLab, GE Healthcare, Chicago, Il) and irradiated with microwaves for 2 h, achieving a final [1-^13^C] pyruvate polarisation of 35%. Subsequently, the sample was dissolved in 38 ml of sterile water, and neutralized with 17.5 g sterile trometamol buffer solution (333 mM Tris and 600 mM NaOH) in 19 ml of sterile water.

The patient was positioned supine with an intravenous (IV) cannula placed in the left antecubital fossa, in a 3T PET-MRI (Biograph, Siemens, Erlangen, Germany) with custom-designed ^13^C clamshell transmit and two (anterior and posterior) 7-channel 1 h/13C receive phased array coils (RAPID Biomedical GmbH).

Sagittal and coronal ^1^H T2 weighted images were acquired for tumour localisation using a turbo spin echo sequence with the following parameters: repetition time = 5000 ms, effective echo time = 80 ms, slice thickness = 5 mm, number of slices = 27, field-of-view (sagittal) = 320 x 320 mm, field-of-view (coronal) = 360 x 360 mm, echo train length = 11, number of signal averaging = 2, fat suppression technique: spectrally adiabatic inversion recovery.

^13^C chemical shift imaging (CSI) was planned as a single slice acquisition (covering the tumour) with slice positioning guided by the ^1^H T2 weighted images and prior MRI and to minimise potential for wrap artefact. The ^13^C receiver bandwidth was centred using a reference phantom containing 1 ml of 8M ^13^C-urea (repetition time = 80 ms, time of echo = 3 ms, flip angle = 10^o^, bandwidth = 10,000 Hz, field-of-view=160 x 160 mm, slice thickness = 30 mm, acquisition matrix = 10×10, reconstruction matrix = 16×16). 40 ml of hyperpolarised [1-^13^C] pyruvate was then injected at 5 ml s^−1^ followed by a 20 ml normal saline flush at 3 ml s^−1^. To account for circulation time 13C CSI measurements were commenced at 25 sec after start of injection. Sequential CSI was acquired every 10 s for 2 min.

^13^C CSI data were analysed (MATLAB 2016; MathWorks Inc., Natick, MA). The individual free induction decays across the CSI grid were apodised with an exponential 5 Hz filter in the time domain and then Fourier transformed. Spectral peak (pyruvate and lactate) areas were then calculated to produce metabolic maps (pyruvate and lactate).

## Results

**Figure 1.**
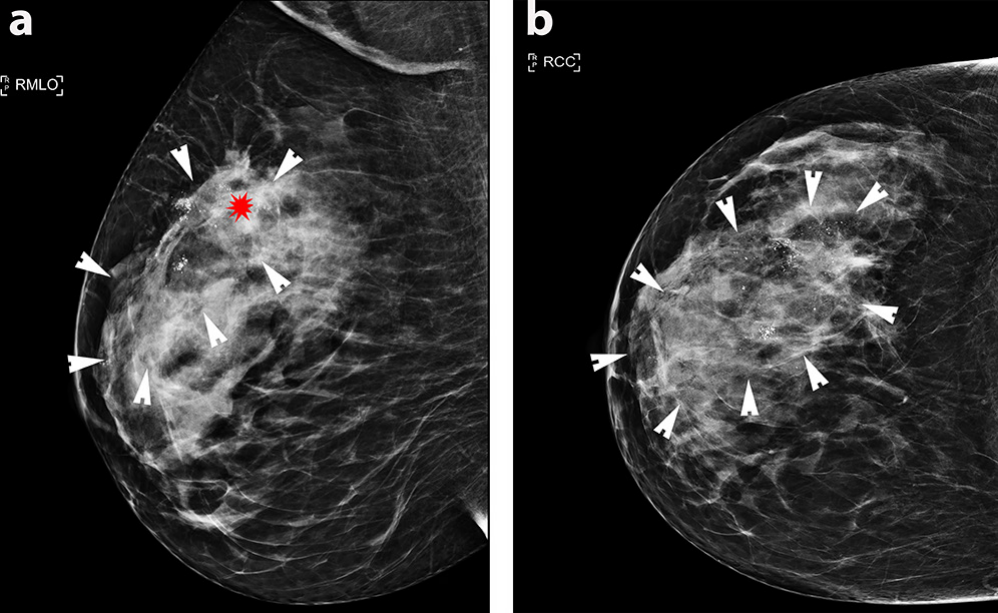
(a)(Medial Lateral Oblique) MLO and (b) (Cranio-caudal) CC mammographic views of the right breast demonstrating extensive malignant micro calcification (outlined with white arrowheads) and an ill-defined mass (red star) on the MLO view.

**Figure 2.**
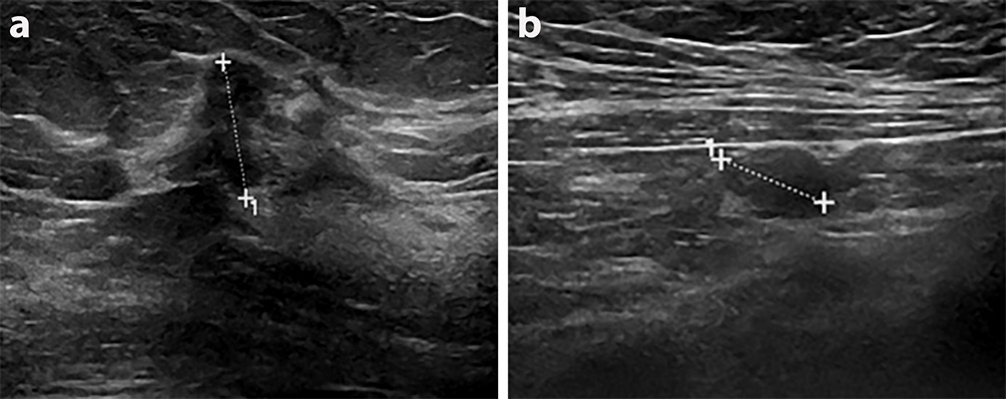
Corresponding sonographic image shows an irregular hypoechoic 2 cm likely malignant mass (a) with likely pathological right axillary lymph node (b).

**Figure 3.**
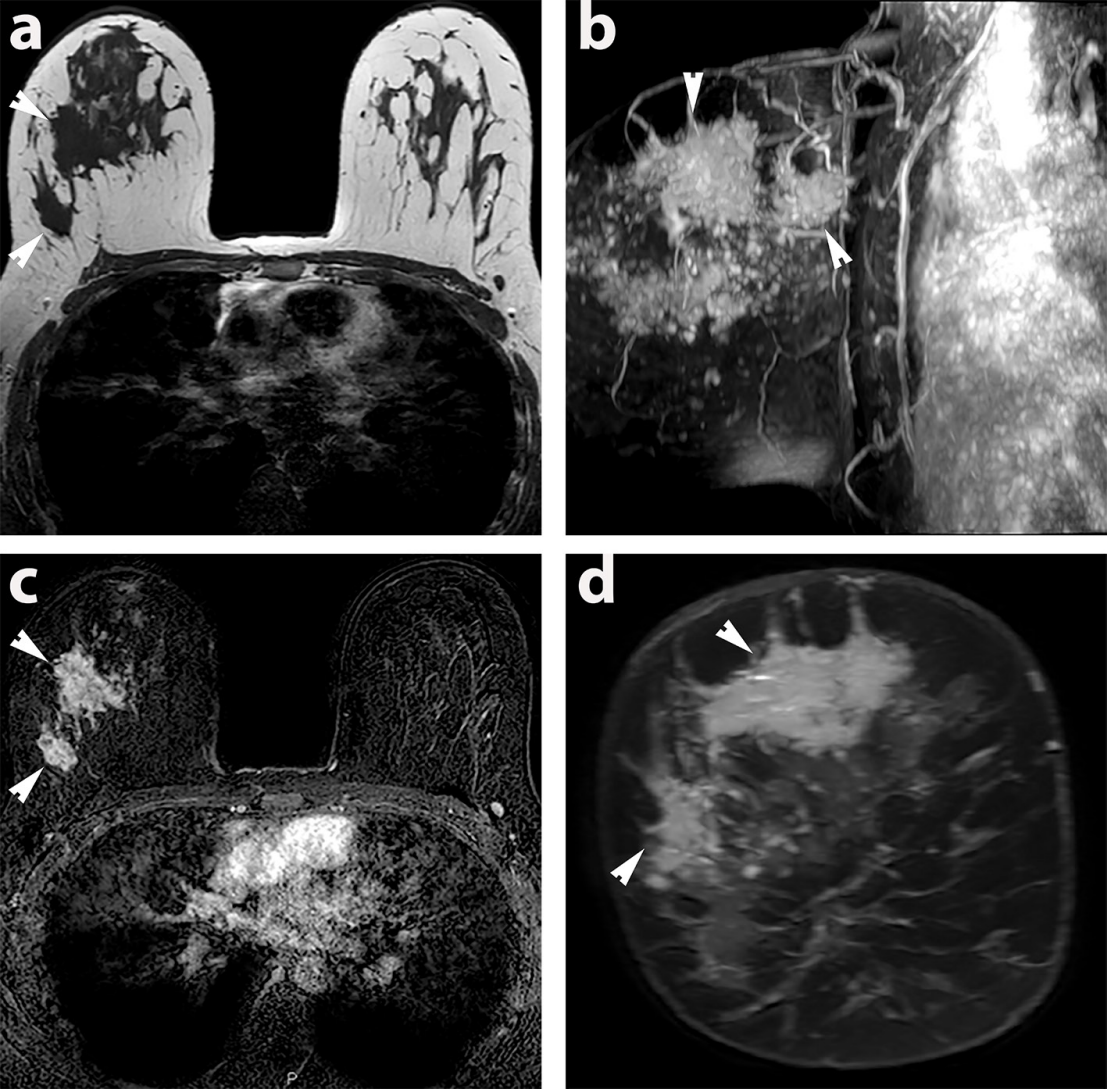
MRI of extensive multicentric right-sided breast cancer in the prone position (a) *T_1_* weighted axial image of upper part of breast (b) 3D MIP sagittal image of the 2 mins contrast enhanced images showing the extent of malignant disease in the breast. (c) 2 min contrast enhanced T1 subtracted axial image of the upper part of the breast (d) T1 post contrast reconstructed coronal image of the tumour. The regions of tumour imaged with HP MRI are highlighted with white arrowheads.

Mammography diagnosed a right-sided 90 mm area of microcalcification within which was an ill-defined mass, the latter only visible on the medial lateral oblique (MLO) view (Figure 1). Ultrasound revealed an irregular hypoechoic mass measuring 20 mm in the right upper outer quadrant, suspicious for malignancy (Figure 2). The right axilla contained a pathological 7 mm lymph node with an obliterated hilum. Dynamic contrast enhanced MRI demonstrated right-sided multicentric disease (*i.e.* tumour extending beyond one quadrant) (Figure 3).

Node positive breast cancer was confirmed histologically via US guided core biopsy of the breast and axillary masses, diagnosing a Grade two invasive ductal carcinoma Luminal A. Stereo guided vacuum assisted biopsy of microcalcification 3 cm remote from the index biopsy revealed high grade ductal carcinoma *in situ* (DCIS), establishing disease extent of 90 mm.

**Figure 4.**
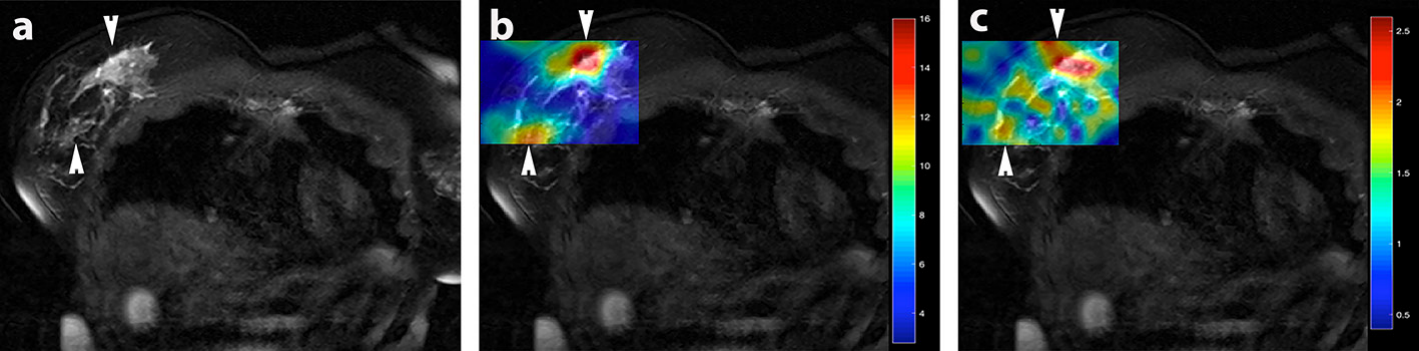
HP-MRI: Coronal *T_2_* weighted MRI with rectangular ^13^C metabolite colour map superimposed. Red denotes regions of highest metabolite concentration whereas blue denotes the lowest. (a) *T_2_* weighted coronal image of the tumour. The regions of tumour are highlighted with white arrowheads. The respective metabolic maps are, (b); pyruvate map, and (c); lactate map. Images are from ^13^C CSI first acquisition.

Figure 4a the *T_2_* weighted coronal localiser image demonstrates the anatomical location of the multicentric tumour within the slice acquired for HP-MRI. The tumour is highlighted with arrowheads. It is locally aggressive and invades the chest wall. Figure 4b shows the distribution of [1-^13^C] pyruvate signal within the breast cancer. Figure 4c demonstrates the corresponding ^13^C-lactate map, showing conversion of pyruvate to lactate via appearance of high signal (red). Maps presented are from the first CSI acquisition (at 25 sec post injection).

**Figure 5.**
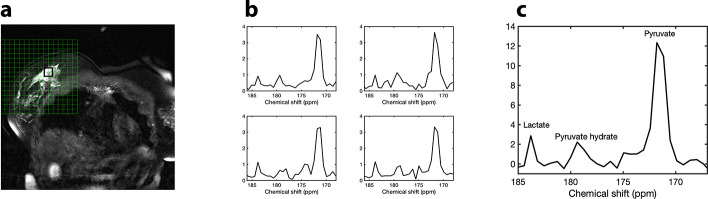
HP-MRI: (a) Coronal *T_2_* weighted image with spectroscopic grid. The black square over the tumour denotes the location of the four spectra presented in (b). (b) Four spectra across the black square in the tumour area acquired 25 sec after injection. (c) Summed spectrum, derived from the addition of the four spectra (b) across the tumour, with the [1-^13^C] pyruvate signal (~171 ppm), the [1-^13^C] pyruvate-hydrate signal (~179 ppm) and [1-^13^C]-lactate signal (~184 ppm).

Figure 5b presents four spectra from the tumour area acquired 25 sec after injection, and Figure 5c the summed spectrum, derived from the addition of the four spectra (Figure 5b) across the tumour, showing the [1-^13^C]-pyruvate signal (~171 ppm), the [1-^13^C]-pyruvate-hydrate signal (~179 ppm) and [1-^13^C]-lactate signal (~184 ppm).

**Figure 6.**
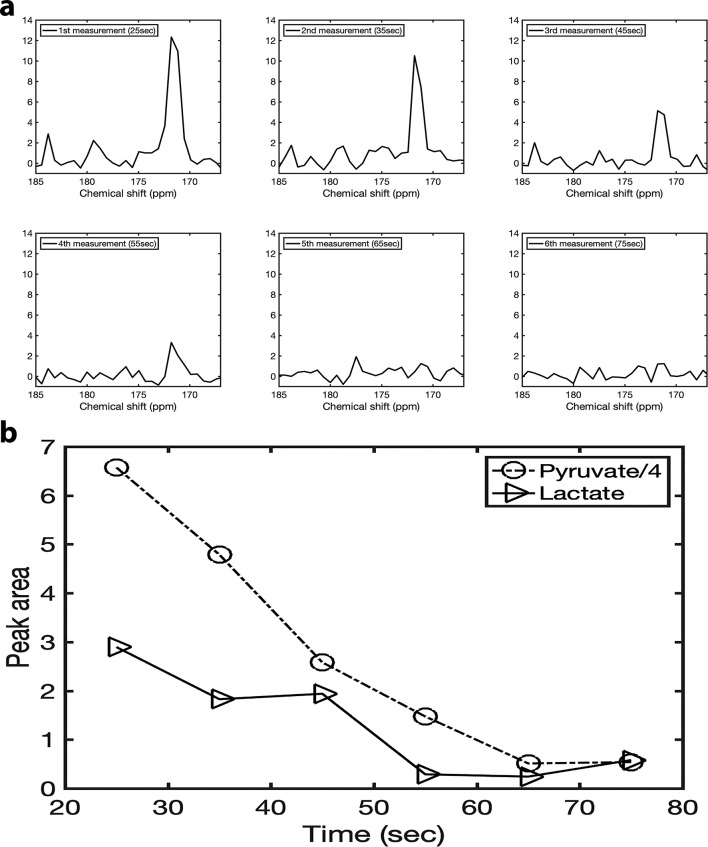
(a) Six summed spectra acquired at six different points after the injection: first measurement at 25 sec, second measurement at 35 sec, third measurement at 45 sec, fourth measurement at 55 sec, fifth measurement at 65 sec, sixth measurement 75 sec. (b) The peak areas of lactate and pyruvate over time. The pyruvate peak area was divided by four for ease of view.

Figure 6 shows the summed ^13^C spectrum from the region of interest (four voxels) in the tumour acquired at six different points after the injection: first measurement at 25 sec, second measurement at 35 sec, third measurement at 45 sec, fourth measurement 55 sec, fifth measurement 65 sec, sixth measurement 75 sec. Figure 6b illustrates the temporal changes of the lactate and pyruvate signal of the summed spectrum during the first six measurements following the [1-^13^C] pyruvate injection.

## Discussion

Cancer cells generate energy via elevated glycolysis with enhanced conversion of pyruvate to lactate, metabolism that is markedly different from normal non-proliferative cells.^4^ Highly glycolytic cancer cells prevent intracellular acidification by excreting the glycolytic end-products lactate and *H* + via the monocarboxylate transporters 1 (MCT1) and 4 (MCT4).^5^ Currently, it is believed that the acidic tumour microenvironment supports aggressive development via excessive growth, increased survival, migration, invasion, and angiogenesis.^1^ Moreover, monocarboxylate transporter (MCT) one is known to be raised in highly proliferative ER-negative breast tumours (compared with ER-positive cases with low proliferation), and, furthermore, is associated with poor survival.^5^ Hence, imaging lactate production via HP-MRI may be a route to identification of aggressive tumours and more accurate outcome prediction.

HP-MRI has already been applied successfully in animal models to assess tumour development and therapeutic response.^6^ In these studies, the metabolic rate of hyperpolarised pyruvate reflects activity of MCT transporters in breast cancer.^7^

We report one of the first cases of in *vivo* non-invasive metabolic assessment of breast cancer using [1-^13^C] pyruvate HP-MRI and correlate the findings with conventional imaging techniques. DCE MRI provided an anatomical map of disease, and is the best generally available method to assess disease extent. However, it depicts breast tissue vascularity, localising increased tumour angiogenesis. As expected, the HP-MRI pyruvate map (which represents delivery via blood flow) appears similar to the DCE MRI image at 2 min. However, areas of increased signal on DCE and HP-MRI pyruvate maps do not occur in tumour exclusively, as normal dense breast parenchyma may also be associated with relatively increased blood flow/volume. The lactate map demonstrates areas where pyruvate was converted to lactate. The highest concentration of lactate is seen within the tumour. There is some signal seen outside the breast on both pyruvate and lactate maps. Signal outside of the CSI region of interest may wrap into this region, but signal within this area will be correctly spatially localised. The metabolite signals that we observe do correspond with the position of the tumour seen on the conventional T2 MRI. Wrap around artefact is a current limitation when scanning the body using the single slice CSI sequence and coil setup. The artefact could be reduced by acquiring images with a dedicated breast coil.

In conclusion, we present a case of a young female with extensive Grade two ductal carcinoma. Her diagnostic work up included all the standard investigations and highlighted the importance of a multimodality approach to accurately map the anatomical extent of disease. The addition of HP-MRI to this case provided proof-of-concept for non-invasive *in-vivo* metabolic assessment of human breast tumour and the potential of identifying aggressive cancers. As this technology is currently in early Phase clinical translation, the information from HP-MRI was not directly used change patient management in this case. However, the patient did elect for a mastectomy and adjuvant chemotherapy. Clinical studies are now needed to determine if ^13^C HP-MRI lactate assessment, as hypothesised by pre-clinical metabolic studies, can indeed differentiate aggressive and more indolent breast tumours in patients. If successful, HP-MRI could become a powerful tool to personalise breast cancer management.

## Learning points

1. HP MRI is a new hybrid technique that allows real-time, non-invasive assessment of cancer cells behaviour. We have shown it is feasible to image breast cancer using HP MRI.
